# Soil microbial CO_2_ fixation rate disparities with different vegetation at a representative acidic red soil experimental station in China

**DOI:** 10.3389/fmicb.2024.1480484

**Published:** 2024-11-21

**Authors:** Chao Long, Zuwen Liu, Renlu Liu, Li Yin, Fuxing Tan, Yian Wang, Genhe He

**Affiliations:** ^1^School of Life Sciences, Key Laboratory of Jiangxi Province for Functional Biology and Pollution Control in Red Soil Regions, Jinggangshan University, Ji’an, Jiangxi, China; ^2^School of Civil and Surveying & Mapping Engineering, Jiangxi University of Science and Technology, Ganzhou, Jiangxi, China; ^3^School of Hydraulic & Ecological Engineering, Nanchang Institute of Technology, Nanchang, Jiangxi, China

**Keywords:** acidic red soil, carbon-fixation, ^13^C stable isotope labeling, *CbbL* gene, structural equation model, vegetation

## Abstract

Soil acidification poses a significant environmental challenge in China’s southern red soil regions, impacting the abundance of soil microbes and their capacity for carbon fixation. The effect of vegetation types on soil’s biological and abiotic components under acidification, and their regulatory role on the CO_2_ fixation mechanisms of soil autotrophic microorganisms, is difficult to examine. This gap in understanding constrains the assessment of the carbon fixation potential of red soils. To address this, indoor cultivation coupled with ^13^C stable isotope labeling was employed to evaluate the disparate abilities of autotrophic microorganisms to assimilate and store CO_2_ across five vegetation soils from the Qianyanzhou acidic red soil experimental station in China. Findings indicate that carbon fixation rates in these soils spanned from 4.25 to 18.15 mg C kg^−1^ soil d^−1^, with paddy field soils demonstrating superior carbon fixation capabilities compared to orchard, coniferous forest, broad-leaved forest, and wasteland soils. The ^13^C fixation rate in the 0–10 cm soil stratum surpassed that of the 10–30 cm layer across all vegetation types. High-throughput sequencing of 16S rRNA, following *cbbL* gene purification and amplification, identified *Bradyrhizobium*, *Azospirillum*, *Burkholderia*, *Paraburkholderia*, and *Thermomonospora* as the predominant autotrophic carbon-fixing microbial genera in the soil. PERMANOVA analysis attributed 65.72% of the variance in microbial community composition to vegetation type, while soil depth accounted for a mere 8.58%. Network analysis of microbial co-occurrence suggested the soil microbial interactions and network complexity changed with the change of vegetation types. Additionally, multiple linear regression analysis pinpointed the Shannon index and soil organic carbon (SOC) content as primary influencers of carbon fixation rates. Structural equation modeling suggested that iron enrichment and acidification indirectly modulated carbon fixation rates by altering SOC and autotrophic bacterial diversity. This investigation shows the spatial dynamics and mechanisms underpinning microbial carbon fixation across varying vegetation types in southern China’s red soil regions.

## 1 Introduction

Soil represents the most substantial carbon store within terrestrial ecosystems, offering vital ecosystem services, including agricultural productivity, water purification, and climate modulation (Anikwe and Ife, 2023). Soil microorganisms are pivotal in upholding soil functionality, thereby sustaining ecosystem services and functional stability, encompassing primary production and nutrient turnover (Banerjee et al., 2018; Bu et al., 2023). Post-industrial human endeavors have precipitated widespread soil degradation, with the world’s agricultural soils forfeiting approximately 133bn tonnes of carbon, potentially impacting ecosystem productivity and atmospheric CO_2_ levels (Sanderman et al., 2017). The principal contributors to soil carbon include plant detritus, root secretions, microbial biomass and necromass, alongside carbon fixation by autotrophic microbes (Mason et al., 2023). These autotrophs, as nature’s quintessential biosynthetic entities, are responsible for an estimated 0.6–4.9 Pg C yr^−1^ of CO_2_ fixation, equating to 25% of total anthropogenic CO_2_ discharges (Bossio et al., 2020; Zheng Z. C. et al., 2022). Exhibiting remarkable adaptability across various ecosystems, they predominantly engage in the carbon cycle via the Calvin–Benson–Bassham (CBB) pathway, facilitated by the rate-limiting enzyme Rubisco (Li et al., 2020). The *cbbL* gene encodes Rubisco I’s large subunit, serving as a marker for carbon-fixing microbes and is extensively utilized to evaluate soil autotrophs’ CO_2_ fixation capacity (Qin et al., 2021; Bu et al., 2023). However, extant research has chiefly concentrated on the influence of land stewardship and disparate soil regions on autotrophic microbial communities and their CO_2_ fixation aptitude (Mukasa Mugerwa and McGee, 2017; Li et al., 2018; Zhou et al., 2019; Bu et al., 2023; Cheng et al., 2023), with a notable dearth of studies examining soil properties’ effects on autotrophic microorganisms and carbon dynamics.

Occupying 36% of China’s arable land, the red soil region is a critical contributor to the nation’s rice production, accounting for over 90% of the output (Huang and Zhao, 2014). Predominantly found in tropical and subtropical zones, these soils are subject to frequent alternations of high temperatures and rainfall, leading to severe erosion and leaching of alkali metal ions. Consequently, this results in elevated concentrations of iron and aluminum oxides, culminating in soil acidification (Xing et al., 2022). The dissolution of stable metal mineral-soil organic matter (SOM) complexes by acid rain further influence of SOM microbial mineralization (Moore et al., 2023). While prior research has concerned the interplay between autotrophic microorganisms, soil attributes, and various factors—including regional, soil type, and agricultural management influences—on these microorganisms’ carbon-fixation capabilities (Chu et al., 2016; Zhou et al., 2019; Wang et al., 2021), the feedback mechanisms and functional behaviors of autotrophic carbon-sequestering microbes in iron- and aluminum-rich acidic red soils remain largely uncharted.

Iron oxides can interact with soil organic carbon (SOC) in multiple ways: they may adsorb, complex with, and co-precipitate SOC, thereby enhancing its stabilization. Conversely, iron-reducing bacteria may facilitate dissimilatory Fe reduction, utilizing SOC and Fe as electron donors and acceptors, respectively, which can accelerate SOC decomposition (Moore et al., 2023; Yao Y. et al., 2023). Moreover, the diversity of vegetation types modulates soil physicochemical properties through processes such as litter decomposition and root exudation, subsequently affecting the composition and diversity of soil protozoa and microbial communities, as well as soil carbon-fixation rates (Bahadori et al., 2021; Bhattacharyya et al., 2022; Spohn et al., 2023; Yao Y. W. et al., 2023; Sun et al., 2024). For instance, Lynn et al. (2017) reported carbon-fixation rates of soil autotrophic microbes in wetland, grassland, and woodland soils to be 85.1, 21.9, and 32.9 mg C m^−2^ d^−1^, respectively. Similarly, Huang et al. (2022) observed notable disparities in carbon-fixation rates between forest and grassland soils in the Loess Plateau. These findings underscore the influence of vegetation types on soil carbon-fixation rates, which are pivotal for projecting the carbon-fixation potential of soil autotrophic microbes in the red soil region. Nonetheless, the underlying mechanisms of this influence remain to be elucidated.

This study seeks to explore the dynamic shifts in CO_2_ fixation rates by autotrophic microorganisms within the red soil region and to furnish a theoretical framework for appraising the carbon-fixation potential of microbes across terrestrial ecosystems in the red soil regions of China. We examined the distribution patterns of soil carbon content and the CO_2_ fixation. Employing bacterial 16S rRNA high-throughput sequencing, real-time fluorescent quantitative PCR, and stable isotope labeling techniques, we quantified the CO_2_ fixation capacities of soil autotrophic microorganisms under different vegetation types. Additionally, we delineated the characteristics of soil autotrophic microbial communities, and identified the key biotic and abiotic determinants influencing carbon fixation.

## 2 Methods

### 2.1 Sample collection and pre-treatment

The research site is situated at the Qianyanzhou Red Soil Hill Comprehensive Development Experimental Station in Jiangxi Province, China, which lies within the subtropical monsoon climate zone. The station experiences an average annual temperature of 17.9°C and receives an average annual precipitation of 1,475 mm. The predominant soil type is red soil, classified as ferralsols, originating from red sandstone and mudstone (Shao et al., 2009; Jiang et al., 2020; IUSS Working Group WRB, 2022; Zhou L. et al., 2024). For this study, soils from five vegetation types were selected: paddy fields (PS), orchards (OS), wastelands (WS), broad-leaved forests (BFS, primarily comprising *Liriodendron chinense, Liquidambar formosana Hance*, and *Cinnamomum camphora Presl*), and coniferous forests (CFS, chiefly consisting of *Cunninghamia lanceolata*, *Pinus elliottii Engelmann*, and *Pinus massoniana*) (Supplementary Figure S1). Following the methodology of Bao (2000), soil samples were collected from three strata: 0–10 cm, 10–30 cm, and 30–50 cm. Utilizing a soil drill and an S-type sampling scheme, five subsamples from each depth were mixed into a single composite sample. This process was replicated for three plots per vegetation type, with each plot separated by a minimum of 200 m, yielding a total of 45 soil samples. Post-homogenization, extraneous materials such as gravel and roots were excised, and the samples were sifted through a 2 mm sieve. Each sample was then bifurcated: one portion first immediately designated for DNA extraction and microbial sequencing, preserved at below −80°C ultra-low temperature refrigerator (DW-HL678D, Zhongke Meiling Cryogenic Technology Co., LTD., Hefei, Anhui, China); the other (remaining sample) reserved as the second part of the research material, air-dried and stored at 4°C refrigerator (BCD-539WT, Haier Smart Home Co., LTD., Qingdao, Shandong, China) for subsequent analysis of soil physical and chemical attributes.

### 2.2 Soil physical and chemical properties analysis

The total iron (Fe) content and its various forms were quantified in 0.2 g of air-dried red soil, which had been passed through a 100-mesh sieve (100 openings per inch). Following the methodologies outlined by Jeewani et al. (2021), different forms of Fe were extracted from the soil samples: free iron oxide (Fe_d_) using the sodium disulfite - sodium citrate - sodium bicarbonate method, amorphous iron oxide (Fe_a_) with ammonium oxalate buffer solution, and complexed iron oxide (Fe_c_) via sodium pyrophosphate solution. Total Fe was extracted by dissolving the soil in a mixture of 5 mL 8 M HNO_3_, 2 mL 12 M HCl, and 2 mL HF. The Fe content in the extraction solution was determined using a flame atomic absorption spectrometer (200 series AA, Agilent, USA).

Soil organic carbon (SOC) was measured employing the external heating method with potassium dichromate. Microbial biomass carbon (MBC) and dissolved organic carbon (DOC) were extracted using the chloroform fumigation-K_2_SO_4_ method and a soil-water mixing procedure at a ratio of 1:5 followed by shaking centrifugation, respectively. The extracts were then analyzed by a total organic carbon analyzer (MultiC/N3100, Jena, Germany). Readily oxidized organic carbon (ROC) was assessed using the 333 mM KMnO_4_ oxidation method. Soil pH was measured using soil-determination of pH-potentiometry method (HJ 962–2018, China) with a pH meter (FE28, Mettler Toledo, USA) at a soil-to-water ratio of 1:2.5. Total nitrogen (TN) and total phosphorus (TP) were quantified using an elemental analyzer (Vario Max CN, Elementar, Germany) and the molybdenum-antimony anti-spectrophotometric method, respectively.

### 2.3 Extraction and high-throughput sequencing of soil DNA

The samples were used for DNA isolation in triplicate. Total DNA was extracted from fresh soil samples utilizing the Power Soil™ Total DNA Isolation Kit (Qiagen, Hilden, Germany), and its concentration and purity were assessed using a NanoDrop nucleic acid quantifier (ND-1000, Thermo Scientific, USA) based on the absorbance ratios of A260/A280 and A260/A230. The DNA samples were subsequently stored at −80°C for future analysis. The carbon fixation functional microbial community was characterized using *cbbL* primers (K2f (5’-ACCAYCAAGCCSAAGCTSGG-3′) and V2r (5’-GCCTTCSAGCTTGCCSACCRC-3′)) and analyzed via the Illumina MiSeq high-throughput sequencing platform (Shanghai Majorbio Bio-Pharm Technology Co. Ltd., Shanghai, China) (Qin et al., 2021). Raw sequences underwent initial processing with Trimmomatic software for filtering and FLASTQ software for assembly. Subsequent quality control, de-noising, merging, and de-chimerization of all sequences were conducted using the DADA2 plug-in within Qiime 2 software. High-quality sequences were then clustered at a 97% similarity threshold into operational taxonomic units (OTUs). The most abundant sequence within each OTU was designated as the representative sequence, with a confidence threshold set at 0.7. Classification of bacterial 16S rRNA and *cbbL* gene sequences was performed against the silva138 and unite8.0 databases, respectively, to ascertain taxonomic information and relative abundance distributions (Tiquia et al., 2002).

### 2.4 Soil carbon fixation culture experiment

To initiate microbial carbon fixation activity, 50 g of dry soil samples, sieved through a 2 mm mesh, were placed into 500 mL conical flasks with rubber stoppers and pre-incubated under illumination at 25°C for 15 days (Ge et al., 2013). CO_2_ was purged from the flasks by injecting a synthetic air mixture (75% N_2_ and 25% O_2_). Subsequently, the flasks containing soil samples were divided into two parallel sets. One set was injected with 0.2 mL of ^13^C-labeled CO_2_, while the control set received 0.2 mL of ^12^C-CO_2_. The CO_2_ concentration within the flasks was maintained at approximately 400 ppm. A 40-day labeling experiment was conducted refer to previous studies (Miltner et al., 2005; Huang et al., 2022), during which air and ^13^CO_2_/^12^CO_2_ were periodically reintroduced every 5 days to maintain oxygenation and constant isotope concentration. Concurrently, ultra-pure water was periodically added to each flask to compensate for soil water evaporation. The culture room temperature was regulated to 25 ± 1°C from 8 am to 8 pm daily, with artificial light intensity sustained at 0.5 mol photons m^−2^ s^−1^; the temperature was adjusted to 15 ± 1°C from 8 pm to 8 am. Post-experimentation, soil samples were air-dried and sieved for ^13^C-SOC content analysis. A 1.5 g air-dried sample was sifted through a 0.15 mm mesh, transferred to a 10 mL centrifuge tube, and treated with 3 mL of 2.5 mol L^−1^ HCl for 24 h to eliminate inorganic carbon. After centrifugation, the HCl was decanted, and the residue was rinsed twice with ultra-pure water to remove residual acid (Ge et al., 2013). The stable carbon isotope ratio (^13^C/C) of the soil samples was determined via isotope ratio mass spectrometry (MAT253, Thermo Fisher Scientific, USA) following HCl treatment. The ^13^C-SOC content and carbon-fixation rate (Rs) were calculated using Equations 1, 2 (Huang, 2021), respectively.


(1) 113C−SOC=SOC×AT%labeled−AT%unlabeled×10

(2) $$R_{S}{=}^{13}\text{CSOC}1/S/T$$

AT% (labeled) signifies the proportion of microbial carbon-fixation isotope atoms present in labeled soil, whereas AT% (unlabeled) indicates the proportion of organic carbon isotope atoms in unlabeled soil. The carbon-fixation rate (Rs) is quantified in mg C kg^−1^ soil d^−1^; ^13^C-SOC denotes the ^13^C-enriched soil organic carbon content, measured in mg kg^−1^. The bottom area of the container (S), with a diameter of 0.05 m, is expressed in m^2^; T denotes the duration of the experiment, which was conducted over a period of 40 days.

### 2.5 Data analysis

Data processing was conducted using Microsoft 365 Excel, statistical analysis was performed with IBM SPSS 19.0, and data visualization was facilitated by Origin 2022. A one-way ANOVA, followed by the Least Significant Difference (LSD) test and Duncan’s multiple range test (DMRT), was employed to discern significant differences in soil environmental factors and soil organic carbon composition across various treatments. The BioProjectID is PRJNA1174083 in SRA database. For the co-occurrence network analysis, only bacterial genera within the top 100 relative abundances were considered, with correlation coefficients exceeding an absolute value of 0.5 and *p*-values below 0.01 deemed statistically robust for network generation. Networks were visualized utilizing Gephi (Barberán et al., 2012), with node size reflecting the number of connections (degree), node color denoting major phyla, and edges indicating pairwise correlations.

Non-metric Multidimensional Scaling (NMDS) based on Bray–Curtis dissimilarity was executed to assess microbial community composition variances, while Analysis of Similarity (ANOSIM) tested the significance of microbial community differences between vegetation types (*n* = 999 permutations). A structural equation model elucidating the relationship between the Rs and soil autotrophic bacterial community, SOC, MBC, Fe, and pH was constructed using IBM SPSS Amos 25.0. In this model, the autotrophic bacterial community was represented by the Shannon index. The model underwent further refinement based on the algorithmic outcomes of the theoretical model, with a *p*-value greater than 0.05 in the chi-square test indicating a satisfactory fit with the data.

## 3 Results

### 3.1 Differences in physical and chemical properties of soil

**Figure 1:**
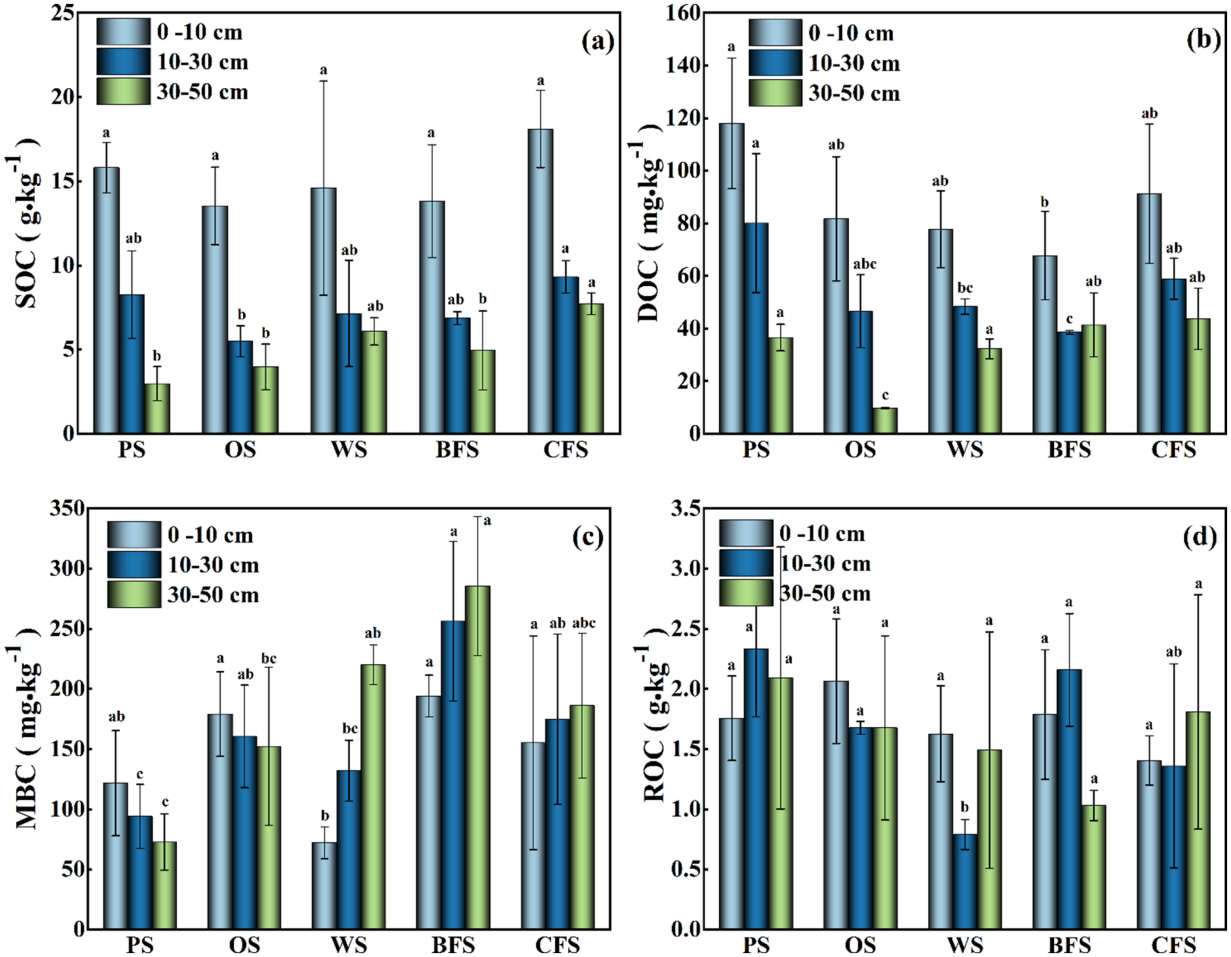
The distribution of **(a)** SOC (soil organic carbon), **(b)** DOC (dissolved organic carbon), **(c)** MBC (microbial biomass carbon), and **(d)** ROC (readily oxidized organic carbon) contents across soils associated with various vegetation types. PS, paddy fields; OS, orchards; WS, wastelands; BFS, broad-leaved forests; and CFS, coniferous forests. Significant differences were indicated by different lowercase letters under different vegetation (*p* < 0.05).

The quantification of soil carbon components under various vegetation types revealed significant findings, as depicted in Figure 1. The CFS exhibited the highest SOC content in the surface layer (0–10 cm), registering at 18.11 g kg^−1^, followed by PS at 15.80 g kg^−1^, WS at 14.58 g kg^−1^, BFS at 13.80 g kg^−1^, and OS at 13.54 g kg^−1^. The highest DOC and ROC contents in the surface layer were found in PS (118.01 mg kg^−1^) and OS (2.06 g kg^−1^), respectively, with the lowest values recorded in BFS (67.77 mg kg^−1^) and CFS (1.41 g kg^−1^). A trend of decreasing SOC, DOC, and ROC contents with increasing soil depth was observed across all vegetation types. MBC content showed a decrease with soil depth in PS and OS, whereas an increase was noted in WS, BFS, and CFS.

TP and TN contents, detailed in Supplementary Table S1, were highest in the surface layer of PS (0.62 g kg^−1^ and 1.93 g kg^−1^, respectively) and exhibited a decline with increasing soil depth across all vegetation types. The soil layers from 0 to 30 cm in PS and OS demonstrated higher carbon, TP, and TN contents compared to WS. Soil pH levels were acidic across all sampling points, ranging from 4.57 to 6.27, with PS soils showing the highest pH values (5.11–6.27) and BFS soils the lowest (4.73–4.79). Furthermore, the contents of Fe_c_, Fe_a_, and Fe_d_ varied between 0.68 to 2.90 g kg^−1^, 0.39 to 2.51 g kg^−1^, and 5.81 to 18.27 g kg^−1^ in Supplementary Table S1, respectively. No significant differences were observed among soil layers for Fe_c_ and Fe_a_ contents. The surface soil layer exhibited lower total Fe content compared to the deeper layers (10–50 cm) in all vegetation types, with the exception of WS.

### 3.2 ^13^CO_2_ fixation rate of soil

Following a 40-day period of indoor cultivation, the ^13^C-SOC content in the soil spanned from 0.75 to 3.21 mg kg^−1^, as detailed in Supplementary Table S2. The ^13^C-SOC content was found to be higher in the 0–10 cm layer compared to the 10–30 cm layer. The ratio of ^13^C-SOC to SOC fluctuated between 0.012 and 0.039%, with the autotrophic carbon fixation contribution to SOC in the 10–30 cm layer being significantly greater than that in the 0–10 cm layer. The ^13^C fixation rates across different vegetation types were ranked as follows: PS (0–10 cm, 18.15 mg C kg^−1^ soil d^−1^; 10–30 cm, 16.71 mg C kg^−1^ soil d^−1^) > OS > CFS > BFS > WS (0–10 cm, 9 mg C kg^−1^ soil d^−1^; 10–30 cm, 4.25 mg C kg^−1^ soil d^−1^). Additionally, the ^13^C fixation rate in the 0–10 cm soil layer was consistently higher than that in the 10–30 cm layer for all vegetation types.

### 3.3 Community diversity of soil carbon-fixation microorganisms

**Figure 2:**
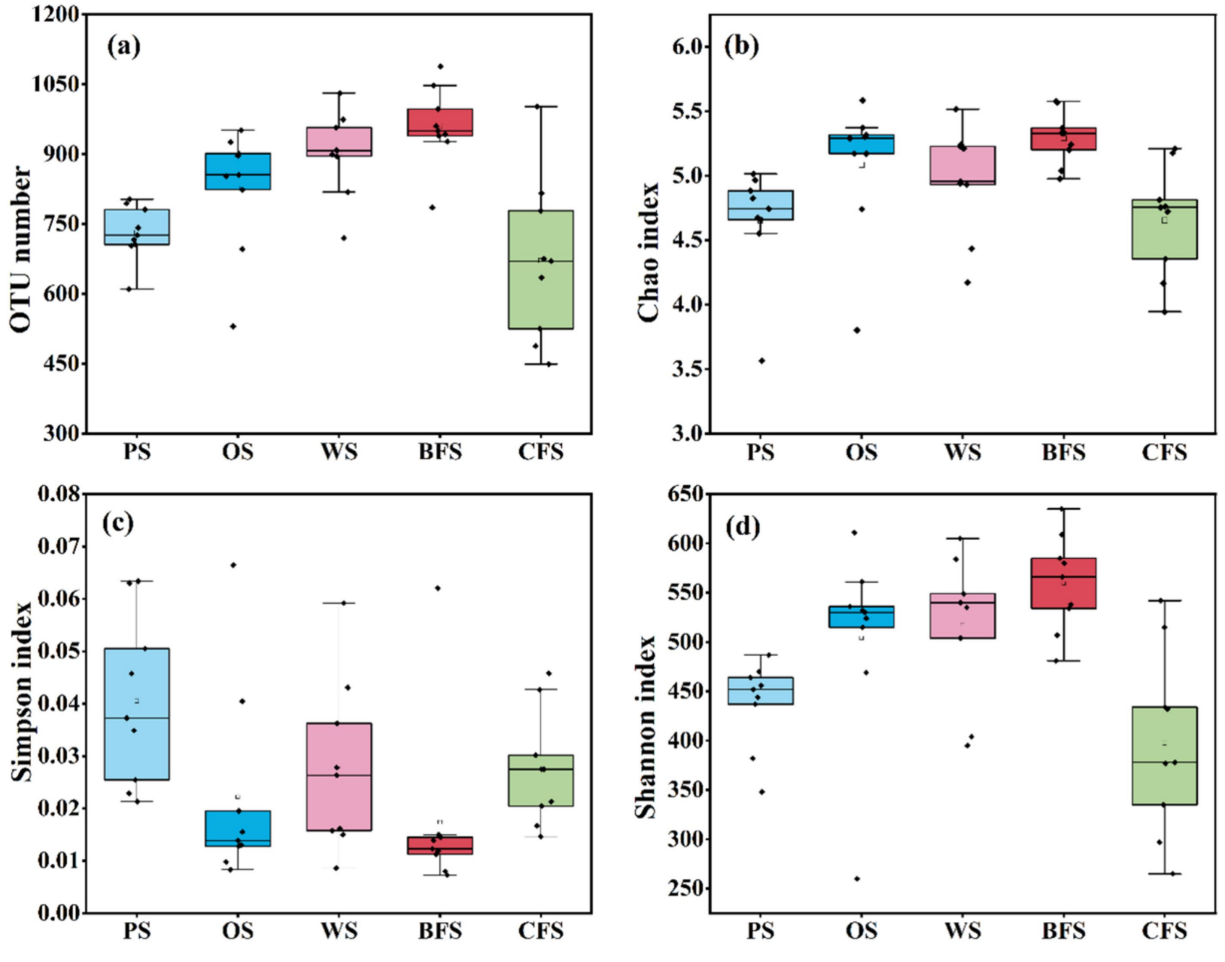
**(a)** OTU number, and **(b)** Chao, **(c)** Simpson, and **(d)** Shannon indices for soil microorganisms across different vegetation types. The sequencing data for microorganisms in various soil layers (0–10, 10–30, and 30–50 cm) are consolidated in the figure, with detailed information provided in Supplementary Table S4.

OTU clustering analysis disclosed a range of 488 to 1,088 OTUs within the sampled soils, as depicted in Figure 2a. Notably, BFS demonstrated a significantly higher number of OTUs in comparison to PS, OS, and CFS. The diversity, evenness, and richness of soil microbial communities, influenced by varying vegetation types, were quantitatively evaluated using several indices: the Chao index for richness, the Simpson index for evenness, and the Shannon index for diversity, with their respective assessments illustrated in Figures 2b–d. The findings indicated that both the Shannon and Chao indices reached their peak values in BFS soil, while the lowest values were recorded in CFS soil. In contrast, the Simpson index was highest in PS soil and lowest in BFS soil. Following a multivariate stepwise regression analysis (Supplementary Table S3) to identify factors influencing carbon fixation rates, the explanation degree of Shannon index for the variability of the synthesis rate of ^13^C-SOC was 67.07%, followed by SOC (35.23%), TP (8.8%), DOC (7.2%), the Simpson index (3.21%), and the Chao index (5.01%). The Shannon diversity index emerged as having the most substantial effect, and this observation is slated for further validation through Pearson correlation analysis.

### 3.4 Soil autotrophic microbial community composition and co-occurrence network

**Figure 3:**
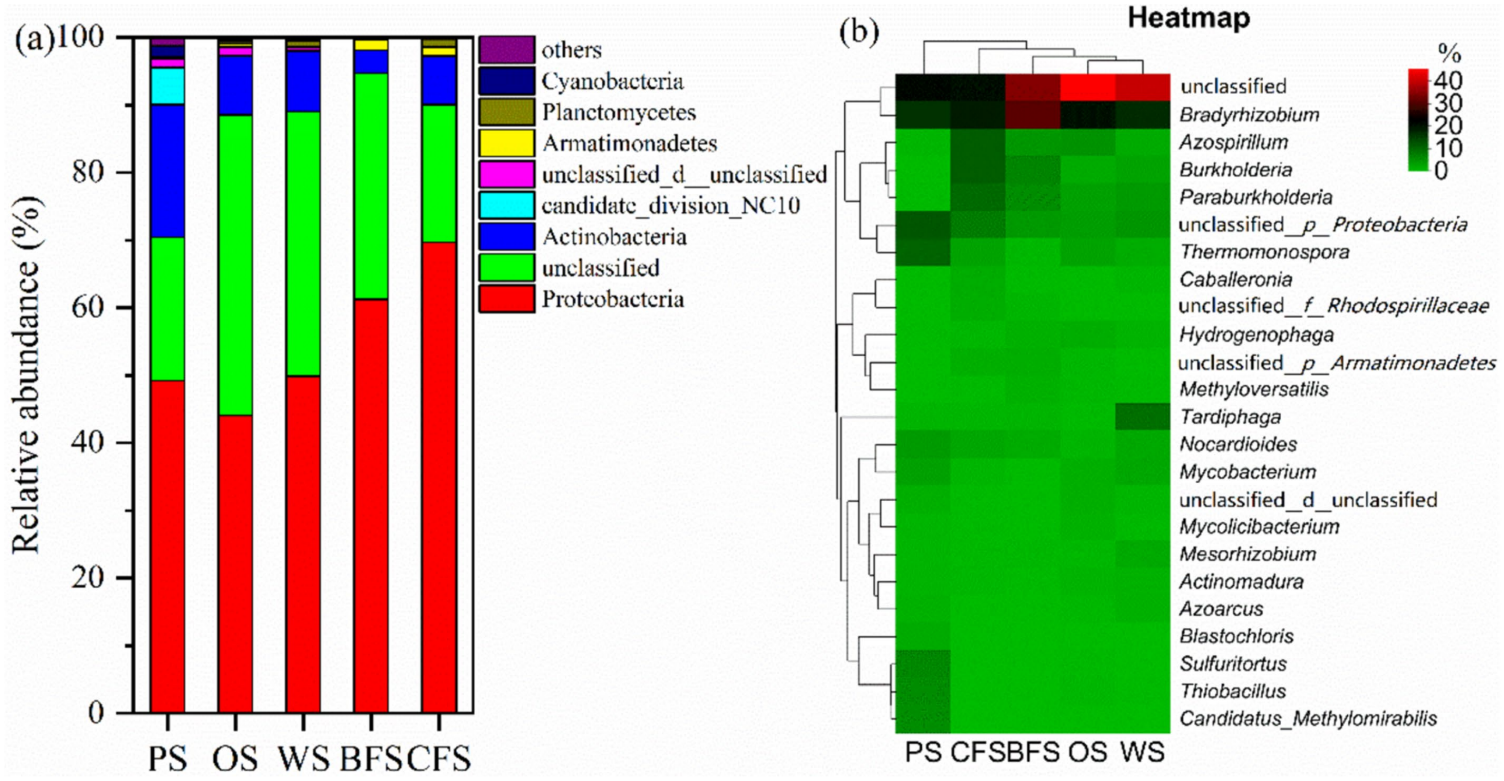
The relative abundance of soil microorganisms at the **(a)** phylum and **(b)** genus levels across various vegetation types. The accompanying heatmap delineates the relative abundance of genera, employing Bray–Curtis dissimilarity and average linkage clustering for both columns and rows; no data transformation has been applied.

Within soil ecosystems, excluding unidentifiable entities, the dominant autotrophs are primarily represented by five bacterial groups: Proteobacteria, Actinobacteria, Armatimonadetes, Planctomycetes, and candidate_division_NC10. Collectively, these groups account for 78.2 to 90.1% of the total bacterial population, as illustrated in Figure 3a. The distribution of Proteobacteria is notably higher in CFS and BFS compared to PS, OS, and WS. In contrast, Actinobacteria exhibit an inverse pattern of relative abundance. At a finer taxonomic resolution, the top 30 autotrophic communities were analyzed, excluding unidentified groups. The genera *Bradyrhizobium*, *Azospirillum*, *Burkholderia*, *Paraburkholderia*, and *Thermomonospora* emerged as the most abundant, as depicted in Figure 3b. *Bradyrhizobium* was the most prevalent across various soil types, with an abundance ranging from 16.1 to 30.0%. *Azospirillum* recorded its highest mutual abundance of 11.1% in CFS. *Paraburkholderia* demonstrated a lower relative abundance in PS, OS, and WS compared to BFS and CFS. Conversely, *Thermomonospora*’s relative abundance was found to be highest in PS, while it was lowest in BFS.

**Figure 4:**
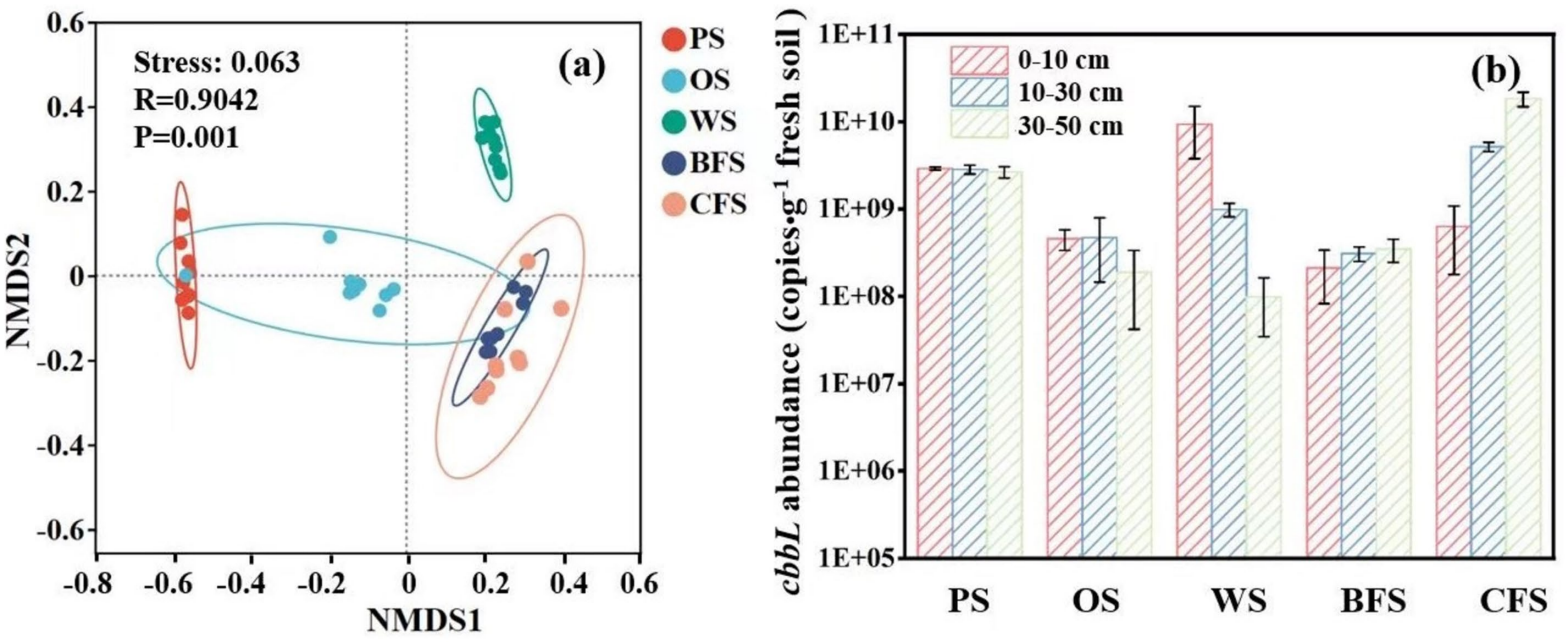
**(a)** OTU-based NMDS analysis; **(b)** the absolute abundance of copies of the soil carbon fixation functional gene *cbbL*.

Non-metric Multidimensional Scaling (NMDS) was employed to discern the driving factors behind the disparities in microbial community structures. The analysis indicated a significant influence of vegetation type on microbial community composition (ANOSIM: R = 0.904, *p* = 0.01), as depicted in Figure 4a. Furthermore, Permutational Multivariate Analysis of Variance (PERMANOVA) attributed 65.72% of the variance to the impact of different vegetation types and 8.58% to soil depths on autotrophic microbial community composition, as detailed in Supplementary Table S5. Quantitative Polymerase Chain Reaction (qPCR) results demonstrated that the copies of *cbbL* functional genes in all vegetation soils spanned from 6.4 × 10^7^ to 1.8 × 10^10^ copies g^−1^ fresh soil (Figure 4b). In PS, OS, and WS, the copies of *cbbL* functional genes diminished with increasing soil depth. Conversely, in BFS and CFS, the copies of *cbbL* functional genes escalated with soil depth, corroborating the ^13^C fixation rate results presented in Supplementary Table S2.

**Figure 5:**
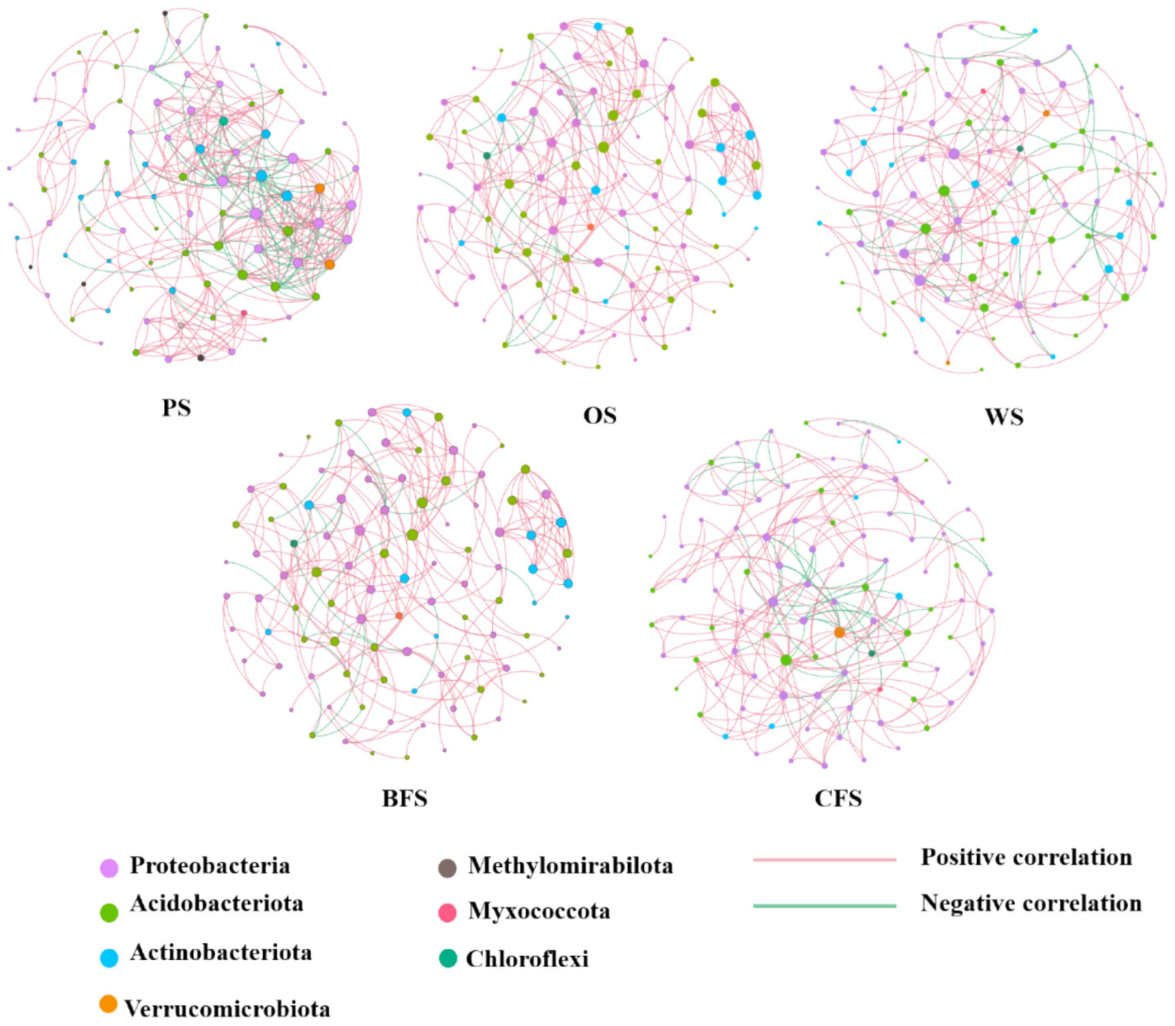
Microbial co-occurrence network analysis among soil microbial communities across various vegetation types.

Co-occurrence networks were generated to elucidate potential interactions among soil microorganisms across five vegetation types. The topological properties of these networks are illustrated in Figure 5. The collinear network associated with PS exhibited the highest edge density, with a network density quantified as 333 edges and a map density of 0.075. In contrast, WS demonstrated the lowest edge density. The average network width size followed the sequence: PS > CFS > WS > OS > BFS, as detailed in Supplementary Table S6.

### 3.5 Correlation between autotrophic microorganisms and environmental factors

**Figure 6:**
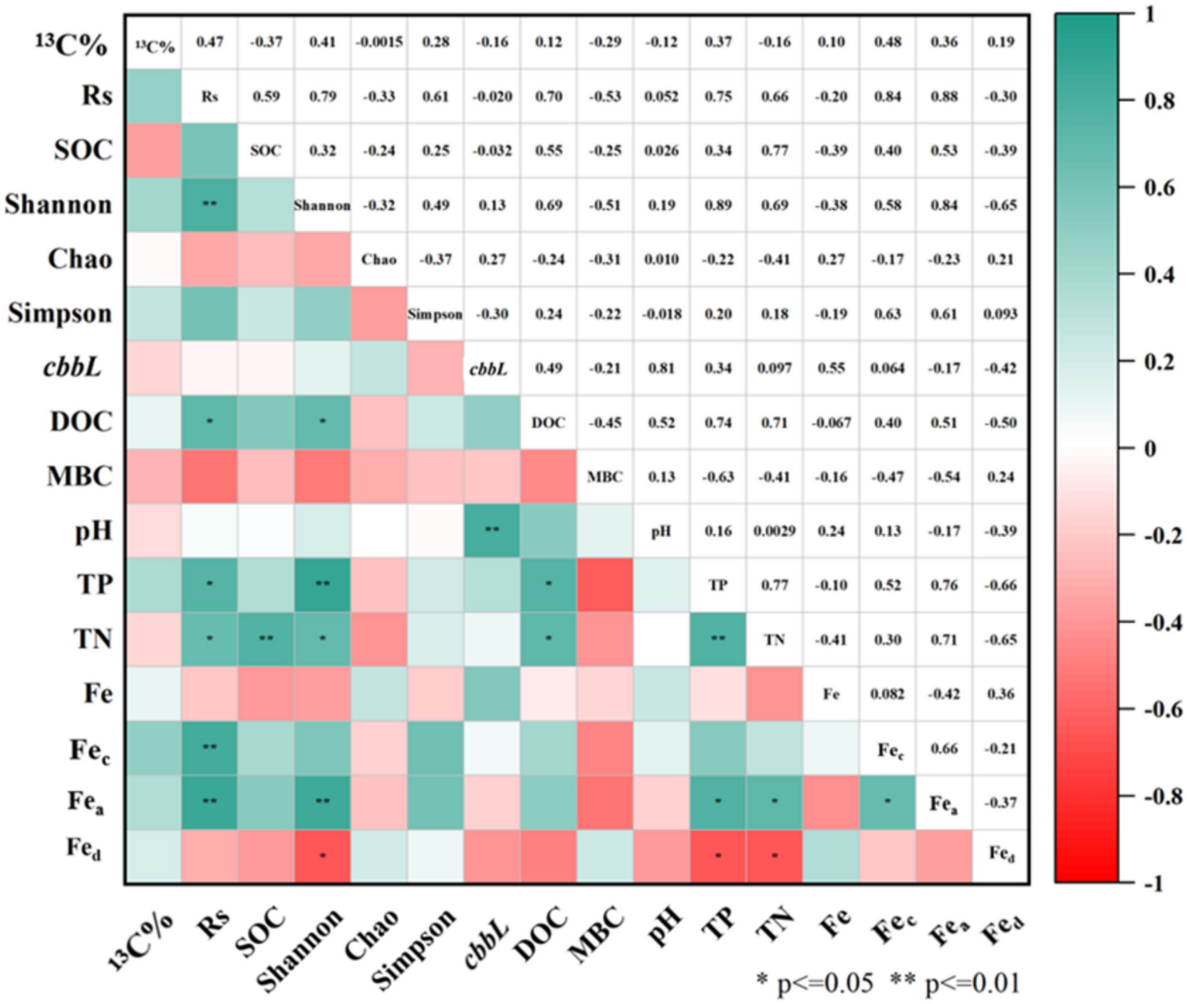
Pearson correlation analysis matrix of soil physicochemical and community diversity parameters.

Pearson correlation analysis (Figure 6) revealed that the soil carbon fixation rate (Rs) was significantly correlated with the Shannon index, TP, TN, DOC, Fe_c_ and Fe_a_ (*p* < 0.05). The Shannon index also showed significant correlations with Fe_a_, Fe_d_, TP, TN and DOC (*p* < 0.05). Additionally, the copies of the *cbbL* gene in soil was significantly correlated with pH (*p* < 0.01). The positive correlation between TP and Rs may be attributed to the role of essential phosphorous compounds in the carbon fixation pathways of autotrophs, including heptose 7-phosphate and D-fructose 1,6-diphosphate in the Calvin cycle, as well as phosphoenolpyruvate in the rTCA and DC/4-HB cycles (Zheng Z. C. et al., 2022). Further Pearson correlation analysis suggested positive associations between SOC, the Shannon index, and Rs, indicating that SOC may enhance facultative autotrophic bacterial growth.

**Figure 7:**
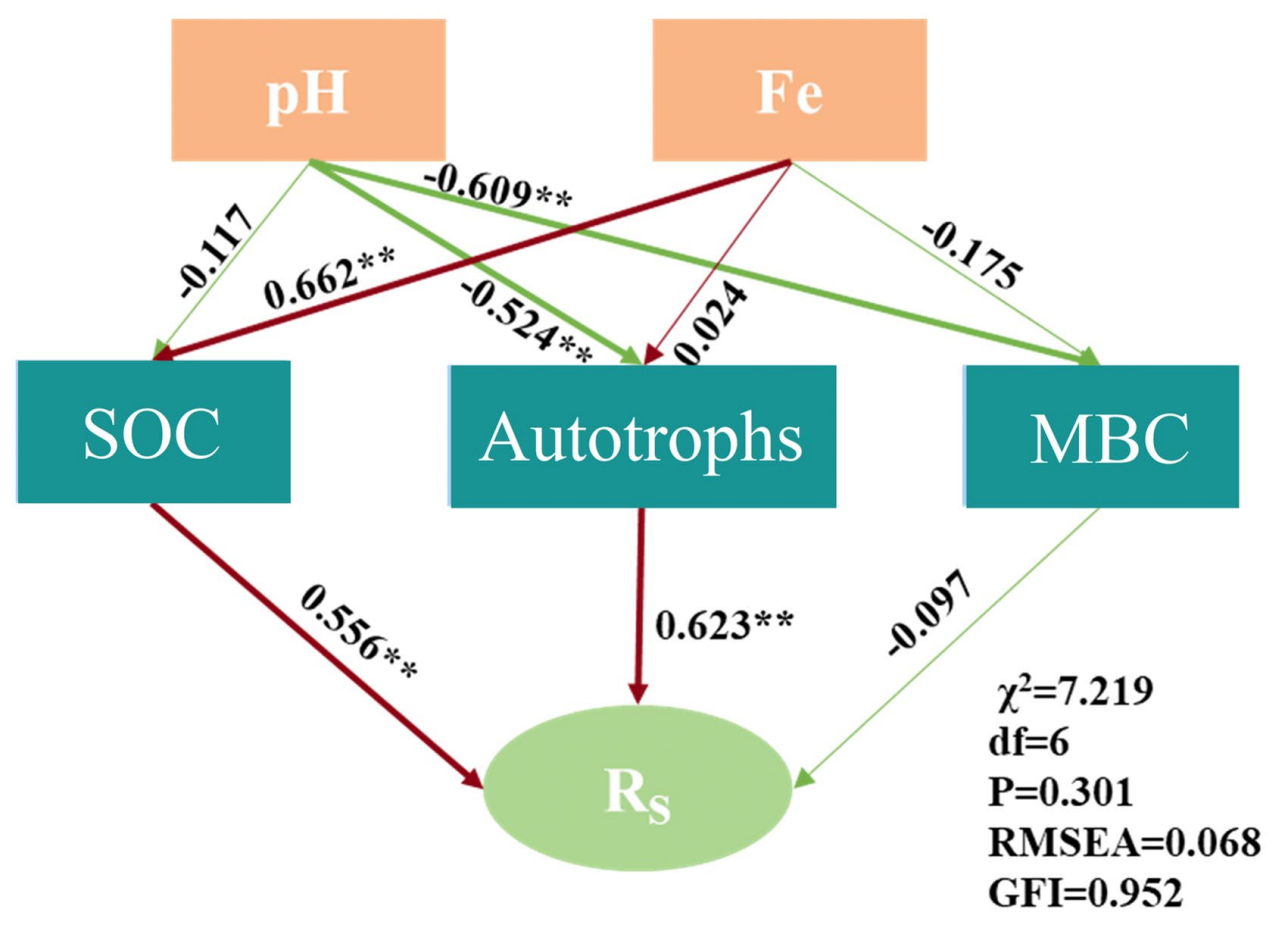
Structural equation model of factors influencing the carbon fixation rate.

The structural equation model (SEM) further substantiated that Rs was influenced by SOC and autotrophs (*χ*2 = 7.219, df = 6, *p* = 0.301, RMSEA = 0.068, GFI = 0.952), as illustrated in Figure 7. The SEM elucidated that Fe indirectly affected the carbon fixation rate by modulating SOC, and pH indirectly impacted Rs by influencing the diversity of autotrophic microorganisms. Consequently, soil acidification may impede the carbon fixation function of bacterial communities (Li et al., 2021).

## 4 Discussion

The surface soil layer of PS and OS exhibited higher SOC, TP, and TN contents compared to other vegetation types, as indicated in Figure 1 and Supplementary Table S1. This disparity may be attributed to the application of inorganic or organic fertilizers, which are rich in nitrogen and phosphorus, on agricultural lands (Hao et al., 2019; Zheng F. J. et al., 2022). Conversely, SOC, DOC, MBC, and TN contents in BFS and CFS were superior to those in WS and OS, which can be ascribed to the enhanced absorption of soil nutrients during plant growth in the initial stages of vegetation restoration in WS (Zhu et al., 2023). As a result, WS exhibited a lower nutrient content relative to other vegetation cover types. The elevated SOC content in BFS and CFS suggests that the input of forest litter may compensate for the nutrient depletion following plant uptake (Pan et al., 2023). However, the humic acid produced by litter decomposition could further reduce soil pH (Rahim et al., 2024). Additionally, the organic acids secreted by coniferous forests, such as *Cunninghamia lanceolat*, to solubilize and assimilate phosphorus from soil minerals, also contribute to the reduction in soil pH (Pan et al., 2023). The highest SOC content recorded in CFS surface soil (18.11 g kg^−1^) might be correlated with the shallow root depth of conifers and the retention of easily degradable rhizosphere sediments within the topsoil (Yan et al., 2018; Duan P. P. et al., 2023). In contrast, broadleaf trees, which possess a higher root biomass, allocate more carbon from decomposable roots to deeper soil strata (Panchal et al., 2022), potentially explaining the observed increase in MBC content in the deeper layer (0–50 cm) of BFS compared to CFS (Figure 1c).

Furthermore, iron plays a pivotal role in the fixation of soil organic carbon (Song et al., 2022). The forms of iron influence the accumulation and stability of soil carbon (Duan X. et al., 2023), where the interaction between iron minerals and organic matter may form an iron-soil organic carbon complex, potentially reducing the bioavailability of soil organic carbon to microorganisms (Herndon et al., 2017; Duan X. et al., 2023; Yang et al., 2023). This interaction may lead to enhanced carbon sequestration in deeper soils. Augmenting the sequestration of soil organic carbon not only ameliorates soil quality and fertility but also contributes to a primary strategy for mitigating global climate change (Nazir et al., 2024).

**Figure 8:**
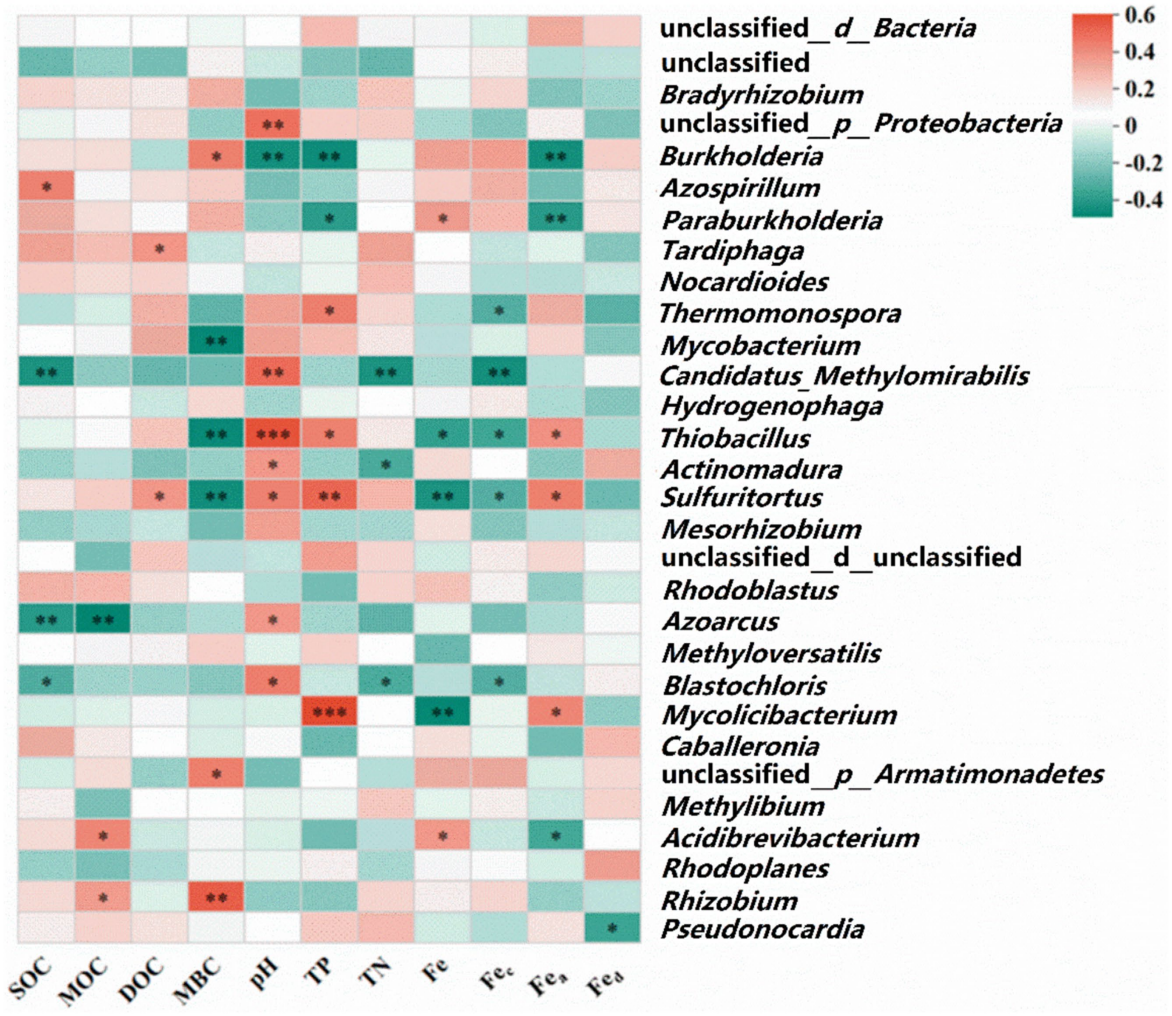
Spearman correlation analysis heatmap between the dominant genus and various environmental factors.

Environmental factors such as temperature, land use, nutrient content, and soil depth are significant determinants of CO_2_ fixation by soil autotrophs (Bhattacharyya et al., 2022; Zhang et al., 2023). Liao et al. (2020) investigated the differences in soil carbon fixation rates between chemically and organically fertilized farmlands, attributing the diminished CO_2_ fixation rate in chemically fertilized soils to decreased soil pH and increased nutrient concentrations. Additionally, Li et al. (2021) posited that alterations in the soil carbon-to-nitrogen (C/N) ratio significantly affect the rate of soil microbial carbon fixation, likely due to the ratio’s facilitation of microbial growth and metabolism, thus enhancing microbial carbon fixation processes. This study demonstrated that PS could sequester more CO_2_, as shown in Supplementary Table S2, while farmland abandonment (wasteland) proved less conducive to soil carbon fixation. PS exhibited a higher rate of carbon fixation compared to other soil types, albeit lower than the CO_2_ fixation rate of Tibetan Plateau soil (18–29 mg C kg^−1^ soil d^−1^) reported by Zhao et al. (2018). Prior research indicates that paddy field soil harbors a larger fraction of obligate autotrophic bacteria and exhibits greater ribulose-1,5-bisphosphate carboxylase/oxygenase (RubisCo) enzyme activity, with its CO_2_ fixation rate being four times that of dryland and forest soils (Lynn et al., 2017; Liao et al., 2020). Numerous studies support the critical role of SOC in modulating carbon fixation rates (Bu et al., 2023), with variations in unstable soil organic matter altering the composition of the autotrophic bacterial community—specifically, the balance between obligate and facultative autotrophs—and thereby influencing microbial carbon fixation potential (Badger and Bek, 2008). Moreover, Fe_c_ and Fe_a_ are significantly correlated with the Rs and positively associated with SOC (Figure 6). Fe_c_ is likely to form complexes with simple SOC and organic acids via coordination bonds (Rezapour et al., 2015), while iron ions may act as electron acceptors for extrinsic iron reduction, affecting carbon fixation within autotrophic bacterial communities (Liu et al., 2019). The Spearman correlation heatmap (Figure 8) demonstrated that eight dominant bacterial species containing the *cbbL* gene were significantly correlated with pH value (*p* < 0.05), likely due to the sensitivity of soil microorganisms, particularly bacteria, to changes in soil pH (Fierer et al., 2007; Raniolo et al., 2023). Moreover, Fe, Fe_c_ and Fe_a_ showed significant correlations with dominant microbial genera (*p* < 0.05). Spearman correlation analysis also revealed significant correlations of TP with *Mycolicibacterium*, *Sulfitobacter*, *Thiobacillus*, and *Thermomonospora*. A global meta-analysis demonstrated that soil pH influences bacterial composition more strongly than spatial or climatic factors (biomes). Furthermore, an analysis of 942 soil bacterial genera found that only 0.8% were tolerant of low pH, whereas 21% were tolerant of high pH. Genera with an acidic pH optimum were more prevalent in humid climates (e.g., boreal forests, and tropical forests) (Zhou X. et al., 2024).

Soil microorganisms play a pivotal role in regulating the carbon budget balance at the soil-atmosphere interface through the assimilation of CO_2_ and the mineralization and decomposition of soil organic matter (Tao et al., 2023; Ding et al., 2024; Wu et al., 2024; Yang et al., 2024). It is commonly posited by researchers that CO_2_ fixation by soil autotrophic microorganisms predominantly occurs at the surface soil-atmosphere interface, leading to a frequent underestimation of the carbon fixation capacity of soil due to the overlooked contribution of deep soil microorganisms (Liao et al., 2023). An observed weak negative correlation between the abundance of the *cbbL* gene across various soil types and their CO_2_ fixation capacity (Figure 4 and Supplementary Table S2) contrasts with findings by Li et al. (2021), who reported a positive correlation between the *cbbL* gene abundance and carbon fixation rates in the Tibetan Plateau and southern red soils. In contrast, Xiao et al. (2018) found no significant association between CO_2_ fixation capabilities and *cbbL* gene abundance in erosive watershed and agricultural soils. This variance may be ascribed to the differing carbon assimilation potentials and metabolic strategies of autotrophs within the soil, as the abundance of autotrophic bacteria does not consistently correlate with CO_2_ assimilation rates (Saini et al., 2011). Multiple linear regression analysis has identified SOC content and the Shannon index as the primary factors explaining variations in microbial carbon fixation rates (Supplementary Table S3). While previous research has indicated that alterations in tillage methods and vegetation succession can diminish microbial community diversity, the contrasting findings regarding community diversity in this study (Supplementary Table S4) may be attributed to the distinct influences exerted by different vegetation types on microbial populations (Zhao et al., 2019; Ramírez et al., 2020; Schmidt et al., 2021).

Obligate and facultative autotrophic bacteria are ubiquitous across all five vegetation types of soil. Certain microbial communities, including Proteobacteria, Actinobacteria, Armatimonadetes, and Planctomycetes, are recognized for their CO_2_ fixation capabilities, as documented in previous studies (Vlaeminck et al., 2011; He et al., 2015; Bhattacharyya et al., 2022). The composition of carbon-fixing bacterial communities is known to vary across ecosystems; for instance, *Rhodopseudomonas palustris*, *Bradyrhizobium*, and *Ralstonia eutropha* are identified as predominant carbon fixation genera in wetlands (Lynn et al., 2017), while *Sulfuritalea*, *Ferriphaselus*, and *Thiohalorhabdus* dominate in karst region soils (Wang et al., 2021). The distinctive iron-rich and acidic conditions of the southern red soil region foster a unique carbon fixation bacterial community (Xing et al., 2022). Microbial co-occurrence network analysis has revealed the development and interactions within ecological niches, such as symbiosis, competition, and predation (Barberán et al., 2012; Hu et al., 2022), as depicted in Figure 5. PS microorganisms exhibit the most intricate co-occurrence network, suggesting that ecosystems with higher nutrient content possess enhanced stability and versatility (Wagg et al., 2019). Conversely, in barren ecosystems, oligotrophic species predominate with minimal predation competition, resulting in weaker interaction relationships. The co-occurrence network displays strong positive correlations and few negative correlations, indicating potential cooperation among microbes to adapt to similar niches (Liang et al., 2017). Facultative bacteria such as *Bradyrhizobium* and *Mesorhizobium* demonstrate versatile capabilities, thriving autotrophically via the Calvin cycle and utilizing energy from inorganic sources like nitrogen, sulfur compounds, and iron oxides (Selesi et al., 2005; Wang et al., 2021). *Thiobacillus* species, for example, are known to use organic substrates as electron donors and Fe as electron acceptors, facilitating dissimilatory Fe reduction, which enhances the microbial carbon utilization rate and strengthens the soil carbon reservoir by increasing the microbial carbon fraction content within the entombing effect (Liang et al., 2017; Yao Y. et al., 2023). Additionally, *Odoricaulis* and *Methylibium* participate in sulfur oxidation processes, while *Bradyrhizobium* forms symbiotic relationships with legumes, contributing to soil organic matter enhancement (Wang et al., 2021).

## 5 Conclusion

This study highlights the intricate interplay between soil carbon dynamics, microbial activity, and environmental factors in red soil ecosystems. The pronounced vertical variation in soil carbon content, which was more significant than horizontal distribution. The carbon fixation rate distribution revealed that PS exhibited nearly twice the fixation rate of WS, underscoring the important role of vegetation type in influencing carbon fixation. Both the Shannon index and SOC were key factors explaining the variability in soil carbon fixation rates across vegetation types. The results indicate that Fe and pH levels modulate carbon fixation by affecting SOC and the diversity of autotrophic bacterial communities. Although autotrophic carbon fixation contributed only 0.012 to 0.039% of total organic carbon within 40 days, its higher contribution in deeper soil layers emphasizes the importance of autotrophic carbon fixation in soils with low nutrient availability. Further research is needed to elucidate the mechanisms by which iron ions mediate microbial carbon fixation and facilitate the formation of stable carbon pools. These findings also have practical implications for sustainable agriculture and soil management. Targeted interventions, such as iron supplementation or pH regulation, could enhance microbial communities and improve carbon fixation. Additionally, this research supports the development of bio-based technologies, including microbial inoculants, to promote carbon storage and mitigate CO_2_ emissions. A comprehensive understanding of microbial processes under different environmental conditions can inform the restoration of degraded red soils and guide climate mitigation strategies through improved soil carbon management. Advanced molecular tools—such as biomarker analysis, metagenomics combined with DNA-stable isotope probing, transcriptomics, and proteomics—will be crucial for identifying the specific microbial processes driving carbon fixation under both controlled and natural conditions. This research can provide a view for understanding the dynamic changes in microbial autotrophic CO_2_ fixation rates in the red soil regions of China.

## Data Availability

The original contributions presented in the study are publicly available. This data can be found here: https://www.ncbi.nlm.nih.gov/bioproject/PRJNA1174083/BioProjectIDPRJNA1174083.
