# Supplementary material for: Soil microbial CO2 fixation rate disparities with different vegetation at a representative acidic red soil experimental station in China
Source: Front Microbiol. 2024 Nov 21;15:1480484. doi: 10.3389/fmicb.2024.1480484 (PMC11619433; doi:10.3389/fmicb.2024.1480484)
Supplement: Supplementary file 1 [file Data_Sheet_1.pdf]

Supplementary Material

Soil Microbial CO_2 Fixation Rate Disparities With Different Vegetation at a Representative Acidic Red Soil Experimental Station in China

Chao Long [1,2], Zuwen Liu [1,2,3], Renlu Liu [1], Li Yin [1], Fuxing Tan [1], Yian Wang [1,*], and Genhe He [1,*]

[1] School of Life Sciences, Key Laboratory of Jiangxi Province for Functional Biology and Pollution Control in Red Soil Regions, Jinggangshan University, Ji'an 343009, Jiangxi, China.

E-mail: Zuwen Liu (liuzw@jxust.edu.cn); Renlu Liu (liurenlu89@163.com); Li Yin (yinli_voyage@126.com); Fuxing Tan (tanfuxing1107@163.com); Yian Wang (nickowya@163.com); Genhe He (hegenhe@jgsu.edu.cn)

[2] School of Civil and Surveying & Mapping Engineering, Jiangxi University of Science and Technology, Ganzhou 341000, Jiangxi, China.

E-mail: Chao Long (lc18832058300@163.com)

[3] School of Hydraulic & Ecological Engineering, Nanchang Institute of Technology, Nanchang 330000, Jiangxi, China.

*** Co-correspondence:**
Yian Wang (nickowya@163.com) and Genhe He (hegenhe@jgsu.edu.cn)

1 Supplementary Figures and Tables

1.1 Supplementary Figures

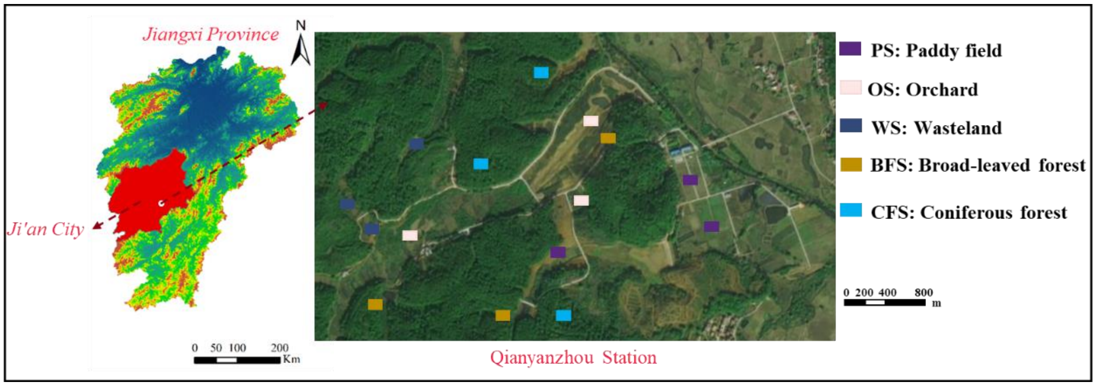

Figure S1. Schematic diagram of the soil sampling locations. Three plots per vegetation type, with each plot separated by a minimum of 200 m.

1.2 Supplementary Tables

Table S1. Soil physical and chemical properties and various forms of iron content.

	Soil depth	pH	TP ($g \cdot kg^{-1}$)	TN ($g \cdot kg^{-1}$)	Total Fe ($g \cdot kg^{-1}$)	Complexed iron oxide (Fe_c) ($g \cdot kg^{-1}$)	Amorphous iron oxide (Fe_a) ($g \cdot kg^{-1}$)	Free iron oxide (Fe_d) ($g \cdot kg^{-1}$)
Paddy field (PS)	0–10 cm	5.11 ± 0.24^b	0.62 ± 0.13^a	1.93 ± 0.06^a	17.79 ± 1.45^c	1.19 ± 0.48^b	2.25 ± 0.67^a	5.81 ± 0.13^c
	10–30 cm	5.62 ± 0.3^a	0.31 ± 0.07^a	0.98 ± 0.30^a	18.25 ± 3.83^b	2.12 ± 0.36^b	2.51 ± 0.47^a	13.28 ± 3.66^a
	30–50 cm	6.27 ± 0.27^a	0.17 ± 0.01^a	0.39 ± 0.03^b	22.43 ± 4.76^a	0.68 ± 0.12^c	0.50 ± 0.64^b	14.44 ± 2.98^b
Orchard (OS)	0–10 cm	5.03 ± 0.66^b	0.27 ± 0.03^b	1.01 ± 0.39^b	21.56 ± 6.37^{bc}	2.29 ± 0.67^a	2.28 ± 0.16^a	14.11 ± 1.03^{ab}
	10–30 cm	4.57 ± 0.33^b	0.32 ± 0.05^a	0.75 ± 0.36^a	22.41 ± 3.23^b	2.25 ± 0.64^{ab}	2.16 ± 0.77^a	13.88 ± 8.35^a
	30–50 cm	4.82 ± 0.51^b	0.20 ± 0.04^a	0.45 ± 0.11^b	25.42 ± 5.75^a	2.32 ± 0.53^a	1.25 ± 0.53^a	18.27 ± 9.98^a
Wasteland (WS)	0–10 cm	5.91 ± 0.22^a	0.16 ± 0.02^b	0.75 ± 0.17^b	31.00 ± 2.65^a	1.71 ± 0.16^{ab}	0.41 ± 0.19^b	12.02 ± 4.23^b
	10–30 cm	5.02 ± 0.81^{ab}	0.16 ± 0.08^b	0.73 ± 0.27^a	27.71 ± 3.16^a	2.90 ± 0.34^a	0.69 ± 0.09^b	13.95 ± 2.45^a
	30–50 cm	5.13 ± 0.21^b	0.19 ± 0.05^a	1.11 ± 0.43^b	24.39 ± 5.77^a	1.76 ± 0.67^b	1.22 ± 0.14^a	13.36 ± 4.98^b
Broad-leaved forest (BFS)	0–10 cm	4.79 ± 0.12^b	0.21 ± 0.07^b	1.20 ± 0.29^b	23.42 ± 3.22^b	2.33 ± 0.82^a	0.75 ± 0.12^b	12.21 ± 0.93^b
	10–30 cm	4.78 ± 0.14^b	0.17 ± 0.02^b	0.77 ± 0.14^a	31.08 ± 7.88^a	2.09 ± 0.12^b	0.39 ± 0.22^b	15.21 ± 6.62^a
	30–50 cm	4.73 ± 0.08^b	0.15 ± 0.02^a	0.60 ± 0.18^b	28.59 ± 5.21^a	2.67 ± 0.33^a	0.52 ± 0.12^b	14.29 ± 1.34^b
Coniferous forest (CFS)	0–10 cm	4.71 ± 0.37^b	0.17 ± 0.03^b	1.21 ± 0.24^b	24.62 ± 2.34^b	2.82 ± 0.60^a	0.55 ± 0.08^b	17.31 ± 3.99^a
	10–30 cm	5.26 ± 0.56^{ab}	0.18 ± 0.06^b	0.99 ± 0.23^a	31.96 ± 2.26^a	2.27 ± 0.12^{ab}	0.84 ± 0.14^b	17.34 ± 4.22^a
	30–50 cm	4.95 ± 0.62^b	0.18 ± 0.05^a	0.66 ± 0.11^b	30.41 ± 9.23^a	2.17 ± 0.45^a	0.85 ± 0.22^b	19.19 ± 6.39^a

Note: the table presents the mean values of three independent experiments. Significant differences were indicated by different lowercase letters under different vegetation ($p < 0.05$).

Table S2. The ^{13}C fixation content and rate, and the ^{13}C-SOC/SOC ratio at each sampling point.

	Soil depth	Paddy field (PS)	Orchard (OS)	Wasteland (WS)	Broad-leaved forest (BFS)	Coniferous forest (CFS)
^{13}C-SOC (mg•kg^{-1})	0−10 cm	3.21±0.43	3.12±0.26	1.59±0.05	2.16±0.15	2.25±0.03
	10−30 cm	2.96±0.39	2.55±0.23	0.75±0.02	1.19±0.01	1.56±0.06
^{13}C-SOC/SOC	0−10 cm	0.018%	0.026%	0.017%	0.012%	0.013%
	10−30 cm	0.026%	0.039%	0.016%	0.017%	0.017%
^{13}C fixation rate	0−10 cm	18.15±2.42	17.61±1.45	9±0.28	12.19±0.84	12.71±0.16
(mg C•kg^{-1} soil•day^{-1})	10−30 cm	16.71±2.18	14.39±1.3	4.25±0.09	6.72±0.08	8.81±0.36

Note: ^{13}C-SOC/SOC is the ^{13}C-SOC fixed by autotrophic microorganisms accounted for the total SOC proportion.

Table S3. Multivariate stepwise regression analysis influencing the carbon fixation rate.

Dependent variable	Explaining variables	Coefficients	S.E.	t.value	P.value	Explained variability (%)
Rs	Constant	-52.121	47.552	-1.096		
	Chao	1.986	1.244	1.596	0.01	5.01%
	Simpson	6.782	1.653	4.101	0.03	6.21%
	Shannon	9.422	8.204	1.148	0.00	67.07%
	DOC	0.014	0.075	0.183	0.05	7.22%
	TP	2.639	15.688	0.168	0.01	8.81%
	SOC	0.351	0.266	1.319	0.02	35.23%
	Adjusted R^2	0.761				

Note: S.E, standard error; Rs, synthesis rate of ^{13}C-SOC; DOC, dissolved organic carbon; Shannon, species diversity index; TP, total phosphorus; TN, total nitrogen.

Table S4. OTU number, and Chao, Simpson, and Shannon indices of soil microorganisms among various vegetation types.

	Soil depth	OUT number	Chao index	Simpson index	Shannon index
Paddy field (PS)	0−10 cm	741.23	444.33	0.0479	4.88
	10−30 cm	772.36	433.00	0.0504	4.40
	30−50 cm	681.05	436.00	0.0232	4.68
Orchard (OS)	0−10 cm	825.08	538.67	0.0195	5.26
	10−30 cm	768.71	438.00	0.0317	4.76
	30−50 cm	884.67	536.00	0.0154	5.23
Wasteland (WS)	0−10 cm	946.85	548.00	0.0167	5.04
	10−30 cm	856.64	449.33	0.0395	4.61
	30−50 cm	901.04	554.67	0.0266	5.23
Broad-leaved forest (BFS)	0−10 cm	946.06	586.00	0.0269	5.08
	10−30 cm	1009.19	574.33	0.0115	5.29
	30−50 cm	924.31	518.00	0.0137	5.50
Coniferous forest (CFS)	0−10 cm	756.36	451.33	0.0205	4.50
	10−30 cm	672.33	400.33	0.0326	4.55
	30−50 cm	584.91	340.00	0.0292	4.91

Table S5. The influence of vegetation type and soil depth on the composition of autotrophic microbial communities was assessed using PERMANOVA, based on the Bray-Curtis distance matrix.

Characteristics	SumsOfSqs	MeanSqs	F.Model	R^2	P.value	P.adjust
Vegetation type	0.6572	0.164	8.021	0.451	0.001	0.002
Soil depth	0.0858	0.043	1.284	0.059	0.265	0.265

Table S6. Characteristic values of co-occurrence network community.

	Average weighting	Diameter of network	Density	Average clustering coefficient	Average path length	Nodes	Edges
PS	7.011	14	0.075	0.582	4.253	95	333
OS	2.404	11	0.049	0.562	4.567	99	238
WS	4.36	9	0.044	0.496	4.426	100	218
BFS	2.562	10	0.053	0.388	4.056	97	245
CFS	4.775	10	0.049	0.494	3.893	98	233
